# Intellectual property on life sciences and biotechnology: state of affairs, impacts and perspectives

**DOI:** 10.1186/1753-6561-8-S4-O48

**Published:** 2014-10-01

**Authors:** Jorge Avila

**Affiliations:** 1 INPI, Rio de Janeiro, Brazil

## 

Everywhere in the world, it has been a major challenge to properly protect inventions and other innovation-oriented achievements in the fields of Life Sciences and Biotechnology. Many aspects make these fields different and probably harder to protect then general inventions on more traditional fields, such as mechanics or chemistry.

First, there are several ethical considerations, which have been taken into account by legislators and judges everywhere. The appropriation of knowledge related to living forms is sometimes seen as illegitimate, particularly that related to the human body. Some legislations ban patents in those fields explicitly. In other cases, courts may rule them unlawful based on ethical considerations not directly linked to any of the traditional patentability criteria. In Brazil, law takes into consideration the rights of traditional peoples on their traditional knowledge, and even on the knowledge that may be developed from genetic resources assumed to belong to traditional communities. This requirements, particularly when they are difficult to accomplish with as they are in Brazil, may stand also in the field of ethical obstacles to knowledge appropriation and commercialization.

A second source of uncertainty in the protection of intellectual assets in those fields has to do with the use of the flexibilities present in the Treaty on Trade-Related Intellectual Property Aspects, generally referred to as "TRIPS". That treaty requires that patents be granted in every technology field but in the field of Life Sciences. With the exemption of genetically modified microorganisms (autonomous, cells not included), living forms may be put out of the general rules for patent granting, and even be non-patentable at all, as it happens in Brazil and in many developing countries.

Last, but not least, the application of the patentability criteria itself for life forms may be trick, leading to conflictive decisions on courts, which may only be pacified in the highest level, as happened recently in the United States.

There are several ways to address the theme of adequate or proper protection. The best approaches, in my opinion, are the ones, which correlate objective and well defined ways of protection to rates or speeds of knowledge disseminations and innovation. Such approaches are applied transversally, among countries or jurisdictions, and may be insightful provided the sample does not comprise countries where jurisprudence on Life-Scieces and biotech patents is not consolidated yet.

Indeed, one of the worst things in terms of the impact of patent systems on innovation seem to be the insufficient consolidation of the understanding of the laws by the courts. Probably that is the reason why many countries follow the North-American example and create specialized courts for IP matters, together with a continuous effort in order to better describe in law the criteria for obtaining a patent. Ethical considerations, for their inherent interpretative nature, probably add to law uncertainty.

Brazil clearly stands in such a bad situation. Several laws rule the appropriation of knowledge and the patentability on Biotech/Biodiversity areas, there is no clear way to fulfill all requirements regarding traditional knowledge, genetic resource definition is unclear and ownership on it is subsequently problematic, as well as on the knowledge which can be developed from it and on the products thus developed. ANVISA "anuência" adds the final touch to uncertainty on those patents when they relate to pharmaceutical products. The result is uncertainty and low investment in all fields related to the Brazilian Biodiversity, and to any pharmaceutical oriented patent. The contradiction between the enormous potential for innovation coming from the spectacular Brazilian Biodiversity and the low rate of product development and patenting may be enough to demonstrate that such uncertainty is clearly detrimental for innovation.

When a country chooses not to protect a particular field of technology knowledge, it clearly avoids investments in that field. It is not clear why Brazilian legislators have decided to ban from patentability insulated natural substances with a known and well defined industrial application. Some say it was due to a perception that there was no endogenous technology capacity, which would be necessary to write the patens and negotiate them. If this is really the case, maybe it is now time to reconsider, not only in Brazil, but also in the countries which have not adopted such protections for the sake of providing no more than the minimum requirements of TRIPS. Of course, minimal requirements are minimal, they are a floor and can never be imagined as a ceiling, as this would confine legislation to the standards established internationally and prevent the country legislators to evaluate and decide with sovereignty the ways the knowledge about the country biodiversity and the results of research on Biotech and Life Sciences should be protected.

In the international comparison, the United States seems to offer adequate conditions for Biotech, to which their patent system helps for sure. Nonetheless, even there some uncertainty results from court decisions, especially when related to genes. This probably suggests that the international community might develop some kind of "sui generis" protection system for segments of genetic sequences with well-defined function and potential industrial application or use.

